# To quantify or not to quantify, that is the question: Semi-quantitative vs. visual analysis of Rb-82 myocardial perfusion imaging PET

**DOI:** 10.1007/s12350-022-02935-4

**Published:** 2022-03-10

**Authors:** Christoph Rischpler, David Kersting, Stephan G. Nekolla

**Affiliations:** 1 Department of Nuclear Medicine, University Hospital Essen, University of Duisburg-Essen, Hufelandstrasse 55, 45147 Essen, Germany; 2 DZHK (German Centre for Cardiovascular Research), Partner Site Munich Heart Alliance, Munich, Germany; 3 Department of Nuclear Medicine, Klinikum Rechts der Isar, Technische Universität München, Munich, Germany

SPECT myocardial perfusion imaging (MPI) remains one of the most commonly performed examinations in the USA and worldwide, with millions of examinations performed per year, and is highly relevant to various questions in coronary artery disease.^1-6^ In addition, SPECT MPI is one of the most commonly used procedures in nuclear medicine in general. There is a large body of data demonstrating the high value of SPECT MPI, particularly its high performance in the diagnosis of coronary artery disease (CAD).^7^ SPECT MPI is of great importance in guiding therapy, i.e., whether a patient should be treated purely with drugs or benefit from revascularization,^6^ and it also has a very high prognostic value in different scenarios.^4,5^ In summary, SPECT MPI is an examination whose value has been demonstrated by a large body of data in thousands of patients. Nonetheless, there are data showing that PET—with several perfusion tracers—is superior for the detection of obstructive CAD compared to SPECT.^8^ In addition to visual and semi-quantitative evaluation of myocardial perfusion, PET allows absolute quantification of blood flow, which can be used to detect microvascular dysfunction or balanced ischemia, offers a reduced exposure to ionizing radiation, and uses very well established attenuation correction algorithms which are especially useful in obese patients.^9,10^ Quantification also represents another independent prognostically relevant parameter.^11^ Another advantage is the independence from technetium generators, for which shortages have occurred regularly in the past. Furthermore, due to the success of PET in oncology more and more nuclear medicine centers have access to this technology, so that PET MPI is an obvious consideration, as it allows a higher utilization of the device with simultaneously better diagnostic significance compared to SPECT MPI. Rb-82 is commonly used as PET MPI tracer because generators are commercially available for this purpose, allowing rapid and straightforward examination of patients within approximately 20 minutes, including stress and rest imaging. It is not surprising that Rb-82 PET has made a significant advance on MPI. The authors of this study have now asked themselves an important question: can the semi-quantitative scores that we know all too well from SPECT MPI be transferred to Rb-82 PET MPI? And then do the semi-quantitative scores obtained agree with a visual analysis? In the present study, these questions were retrospectively investigated in 108 patients who underwent Rb-82 PET MPI. The data were then analyzed semi-quantitatively, and the parameters for ischemia established from SPECT MPI were applied to the data. These data thus collected were then compared with visual analysis. The authors demonstrated that the established limits of an abnormal SPECT MPI for ischemia, namely a summed stress score (SSS) ≥4 in the presence of a co-occurring summed difference score (SDS) ≥2, were very sensitive with respect to the presence of visually detectable ischemia. Indeed, it was the case that all but one of the 31 visually detectable areas of ischemia diagnosed were detected. On the other hand, of the 55 total semi-quantitative analyses considered positive, 25 were considered negative for ischemia on visual analysis. An interesting subgroup analysis, namely those patients with low to intermediate pretest probability, which at the same time represented with 77 subjects the largest group of patients, revealed that the minimal SDS of visually ischemic MPI PETs was 4. The authors concluded that in this patient population, a purely semi-quantitative analysis might be sufficient, which in turn could reduce the work and analysis time spent per scan. Last, a follow-up analysis was also performed, albeit over a small duration of one year. In patients who experienced any of the events consisting of death from a possible cardiac event, invasive coronary angiography, PCI, or CABG, semi-quantitative analysis either agreed with visual analysis or was positive with normal visual analysis. Thus, the case of an event occurring with negative semi-quantitative analysis concurrent with positive visual evaluation did not occur, again indicating a very high sensitivity of the established semi-quantitative scores of MPI-SPECT on Rb-82-PET.

While these are first very valuable evidences that the semi-quantitative scores from SPECT MPI can be transferred to Rb-82 PET MPI for a highly sensitive evaluation with relatively high confidence, some open questions still arise. For the analysis, the authors used a standard normal database supplied by the manufacturer of the software. It can be speculated that the results would have been better if an in-house normal database had been established. Furthermore, as is unfortunately often the case in CAD studies, only a fraction of patients received invasive coronary angiography to assess coronary status, thus lacking a reference standard and limiting the interpretability of the data. In addition, the follow-up was short, and the question of prognostic significance can thus only be answered to a very limited extent and also in view of the relatively small patient population. Nevertheless, this study again highlights the strengths of an objective evaluation of nuclear perfusion imaging using semi-quantitative evaluation. And this even works comparatively well with very widely available analysis software and without the large efforts of quantitative analysis and building an in-house normal database. Moreover, with the advent of PET systems using digital detector technology in various centers a further increase in image quality and quantification accuracy is possible– with first results pointing at benefits also for PET MPI.^12^ This study used data from a conventional scanner and the selectivity for detection of ischemia might even be improved with future technologies. This again highlights the power and increasing indispensability of quantification in cardiac perfusion imaging. Thus, the answer to the question posed in the title of our editorial is clear: Quantify!
